# Effects of the speed on the webbed foot kinematics of mallard (*Anas platyrhynchos*)

**DOI:** 10.7717/peerj.15362

**Published:** 2023-05-15

**Authors:** Dianlei Han, Hairui Liu, Zhiqian Tong, Jiahang Pan, Xinzhong Wang

**Affiliations:** School of Agricultural Engineering, Jiangsu University, Zhenjiang, Jiangsu, China

**Keywords:** Mallard foot, Webbed foot kinematics, Joint angle, Gait

## Abstract

In this study, the effect of the speed on the webbed foot locomotion of the mallard was analyzed based on a considerable number of reliable indoor test data. Four adult male mallards were selected for analysis, and the locomotion speed of the mallard was controlled using the treadmill at an accurate and adjustable speed. The locomotion pattern of the webbed foot of the mallard at different speeds was recorded using a high-speed camera. The changes in the position and conformation of the webbed foot during locomotion on a treadmill were tracked and analyzed using Simi-Motion kinematics software. The results indicated that the stride length of the mallard increased, and the stance phase duration was shortened with the increase of the speed, whereas the swing phase duration did not vary significantly. The duty factor decreased with the increase of the mallard speed but not drop below to 0.5, because the mallards flew with their wings, or moved backward relative to the treadmill with the further increase of the speed. Using the energy method to further distinguish gait, and through the percentage of congruity analysis, it was found that between 0.73 and 0.93 m/s, the gait experienced a transition from walking to grounded running, with no significant changes in spatiotemporal parameters. At speeds between 0.93 and 1.6 m/s, mallards adopt a grounded running gait. The instantaneous changes of the tarsometatarso-phalangeal joint (TMTPJ) angle and the intertarsal joint (ITJ) angle at touch-down, mid-stance and lift-off concomitant with the change of the speed were examined with the TMTPJ and ITJ angle as the research objects. Moreover, the continuous changes of the joint angles were examined in a complete stride cycle. The result indicated that the increase of the speed will also make the TMTPJ and ITJ angle change ahead of time in a stride cycle, proving the shortened stance phase duration. The ITJ angle changed much more than the TMTPJ. Thus, the above result reveals that the mallard primarily responds with the increase of the speed by adjusting the ITJ, instead of the TMTPJ. The vertical displacement of the toe joint points and the toe joint angle was studied (α joint angle is between the second toe and the third toe; β joint angle is between the third toe and the fourth toe) with a complete stride cycle as the research object. The distal phalanxes of the second, third and fourth toes first contacted the ground, and the proximal phalanx touched the ground in turn during the early stance phase duration of the mallard, as indicated by the result of this study. However, the toes got off the ground in turn from the proximal phalanxes when the mallard foot got off the ground. With the decrease of the interphalangeal α and β joint angles, the foot web tended to be close and rapidly recovered before the next touch-down. The above result reveals that the webbed foot of the mallard is a coupling system that plays a role in the adjustment of speed.

## Introduction

Birds show superiorities in terms of migration, predation, and escape from the threat of natural enemies, in which the solid hind limbs take on a critical significance. Birds will change their gaits with the increase of their speed. Mallard ducks are the ancestors of all domestic ducks except Muscovy ducks. It commonly inhabits soft shore areas (*e.g*., rivers, lakes, and beaches). In particular, there are two webs among the toes in the mallard foot. Little research has been done on the ground movement characteristics of this semi-aquatic bird.

van Coppenolle & Aerts (2004) studied the spatiotemporal parameters (*e.g*., stride length, stance phase duration, swing phase duration, and duty factor) of the hind limbs of the white stork using high-speed cameras and other equipment. Their research has suggested that although the above-mentioned slender-legged stilt birds walk with similar dynamics to other bipeds, slender legs do limit their walking speed, with a possible aim to avoid excessive musculoskeletal stresses. Verstappen & Aerts (2000) studied the spatiotemporal parameters of the black-billed magpie using high-speed cameras. They confirmed that swing phase duration is independent of speed. The gaits shifted from walking to running or jumping at the speed of 1 m/s, and the latter was the first choice. To distinguish between walking and grounded running, it is necessary to assess the fluctuations of gravitational potential energy and kinetic energy of the body’s centre of mass (Gatesy & Biewener, 1991; Stoessel & Fischer, 2012; Nyakatura et al., 2012; Andrada et al., 2013). In running, the kinetic energy of forward motion (horizontal kinetic energy, E_kh_) and the sum of the gravitational potential energy (E_p_) and vertical kinetic energy (E_kv_) are in-phase, whereas in walking, an out of phase exchange of these two energies occurs (Cavagna, Heglund & Taylor, 1977). The percentage of congruity (%Congruity), as proposed by Ahn & Biewener (2004) more accurately compares the form of the graphs of two energies and is therefore suggested to reflect the phase relationship better than just comparing local minimum values in the phase calculation (Nyakatura et al., 2012). Andrada et al. (2015) studied quail, oystercatcher, northern lapwing, pigeon, and avocet locomotion gaits using an X-ray camera and treadmill, and their birds neither low-speed walking nor high-speed running. Birds employ mixed gaits (*e.g*., one-step walking followed by one step using running mechanics) more often than walking. A minimalist pronograde virtual pivot point model was built by simulating quail’s gait. Using high-speed cameras and treadmills, Rubenson et al. (2004) demonstrated that the ostriches’ selection between an inverted-pendulum walking gait and grounded running minimizes the metabolic-energy costs of locomotion. This study significantly supports the argument that minimization of metabolic-energy costs is a vital determinant of the gait selection of terrestrial animals. Verstappen, Aerts & van Damme (2000) studied the limb joints of the black-billed magpie in three gait patterns of walking, running and hopping under a high-speed camera. Their findings revealed that the extension and flexion patterns of the joints were comparable across gaits. The magpie prefers a hopping gait. The hindlimb kinematic patterns are elucidated as follows. Throughout the continuous knee flexion, the leg continues to move relatively backward, while the hip and ankle joints are extended. Flexion of the ankle and hip joints aids in foot lift. Magpie hindlimb locomotion patterns are comparable to those of other terrestrial and flying birds, while substantial variances at internal toe angles in humans. The variation in the configuration and morphology of their legs might explain this discovery (Verstappen, Aerts & van Damme, 2000). They have investigated the external and internal toe angle between the magpie’s hallux and third toe, instead of the second and fourth toe.

The regularity of joint angle changing with speed has been extensively studied. The main joint angles comprise tarsometatarso-phalangeal joint (TMTPJ), intertarsal joint (ITJ), knee joint, hip joint, and so forth. Zhang et al. (2019) studied the spatiotemporal parameters, instantaneous and continuous changes of the TMTPJ and ITJ angles of pheasant using high-speed cameras, treadmill and Simi-Motion software. Their results indicated that TMTPJ joint angle was not to be significantly affected by changes in speed, but changed over larger ranges than the ITJ angle. Stoessel & Fischer (2012) explored the biped motion of the *Eudromia elegans*, *Coturnix coturnix* and *Corvus monedula* by biplane high-speed x-ray video analysis. Birds show similar limb proportions. Even small changes in limb proportions and hip heights may result in differences in joint angles between birds and even among birds of the same species. Andrada et al. (2013) studied the hind limbs of quails using X-ray, high-speed camera, Simi-Motion software, and other equipment. As indicated by their results, quails adapt to ground motion by adjusting the whole or local locomotion of the hind limbs. Nearly all elastic deformation occurs in the ITJ from the joint perspective. In quail (Reilly, 2000), the femur does not move during locomotion, and the TMTPJ serves as a major moving joint that significantly contributes to the increase of limb length at the propulsive phase of the stride. Thus, the quail increase their speed by moving the limb faster and farther. Kambic, Roberts & Gatesy (2014) studied guinea fowl from the perspective of coronal plane using an X-ray and high-speed cameras. They revealed that the transverse position of the foot is adjusted by the rotation of the long axis of the femur (up to 38°), and the swing of the foot is readjusted by the rotation of the long axis of the tibia (up to 65°). The rotation of the tarsometatarsal long axis is minimal, and the abduction/adduction of the hip, knee, and ankle joints is minimal. Nyakatura et al. (2012) studied the changes of spatiotemporal parameters and the changes of continuous and instantaneous joint angles in a stride cycle of the northern lapwings with X-ray, high-speed camera, Simi-Motion software, and so forth. The results suggested that the supple posture of hind limbs and the grounded running gait serve as an evolutionary constraint for walking birds, and the efficient use of grounded running can enable walking birds to adapt to higher speeds more smoothly. Zhang et al. (2018) studied the locomotion of ostriches on hard ground and sand ground using high-speed camera equipment and kinematics software. Their result revealed that there is no significant difference in the changes of toe joint angle when ostriches walk or run on sand ground, and the third toe and fourth toe are coupled as a whole with the third toe as the primary load-bearing element with the fourth toe as the complementary load-sharing element to mainly ensure the lateral stability of the permanently elevated metatarsophalangeal joint (Zhang et al., 2017). The above-mentioned research has primarily introduced the changes of birds’ gait and main joint angle, which provides references for the methods and ideas of this study.

Usherwood, Szymanek & Daley (2008) established an extremely simplified compass gait model to investigate the walking-running transition speed of humans and ducks. Ducks walk as inverted pendulums, and their legs swing almost passively, with a relative velocity of nearly 0.5, well consistent with the theoretical model. Nudds et al. (2010) investigated the barnacle geese and found that the gait conforms to the classical pendulum mechanics-based model of walking. Besides, no evidence of a gait change was found. Barnacle geese are mechanically and energetically inefficient walkers relative to more specialist cursorial birds since they compete selection pressures for swimming and flight. Nevertheless, their upper walking speed is limited by morphology (*via* kinematics) rather than metabolic capacity (energetics). Provini et al. (2012) studied the land motion and water motion gait of the semi-aquatic ringed teal (*Callonetta leucophrys*) using high-speed cameras, ray cameras, chemical markers, and so forth. As revealed by their result, the muscles and bones correlated with motion in the two environments with different physical characteristics will undergo morphological changes to adapt to the varying environment. Abourachid (2000) has suggested that postural differences between Mallard ducks and Indian runners affect kinematic characteristics. The strategies for increasing speed are different in the two species as follows. Mallard ducks increase the amplitude of locomotion, which is consistent with other non-running birds, whereas Indian runners increase the frequency of locomotion, which is consistent with cursorial birds. Thus, interspecific differences in locomotor traits in birds may be a functional response to the physical environment, and they are likely to be supported by morphological adaptations. Taylor-Burt & Biewener (2020) studied the hind limb kinematics and muscle function of the mallards during vertical take-off on land and in water. They highlighted that mallards may be challenged to tune their muscle properties for locomotion across differing environments. The above scholars have primarily studied and analyzed the gait and hind limb locomotion patterns of semi-aquatic birds, such that a foundation is laid for further research to choose mallard ducks as research objects.

Although some significant research on mallard bipedal locomotion has been conducted, there has been rare potential or detailed research on the webbed foot kinematics. Table 1 lists the current research contents on avian leg kinematics. The results and regularity on the spatiotemporal parameters, joint angle changes, and toe vertical displacement of the mallard foot were studied using high-speed cameras and treadmills to reveal the effect of the speed on the main joint angle and the change pattern of toes and webs motion. As such, our data may provide insights into the semi-aquatic birds using one or two joints during ground movement to accommodate the increase in speed.

**Table 1:** The current research contents of avian leg kinematics in our references.

Research contents	Species	Data collected
Locomotion gait	Mallard	Usherwood, Szymanek & Daley (2008), Abourachid (2001)
	White stork	van Coppenolle & Aerts (2004)
	Black-billed magpie	Verstappen & Aerts (2000), Verstappen, Aerts & van Damme (2000)
	Quail, Oystercatcher, Northern Lapwing, Pigeon, and Avocet	Andrada et al. (2015)
	Ostriches	Rubenson et al. (2004), Zhang et al. (2018)
	Pheasant	Zhang et al. (2019)
	Barnacle geese	Nudds et al. (2010)
	Ringed teal	Provini et al. (2012)
	Indian runner	Abourachid (2000), Usherwood, Szymanek & Daley (2008)
	Aylesbury duck	Usherwood, Szymanek & Daley (2008)
	Northern lapwing	Nyakatura et al. (2012)
Joint angle	Pheasant	Zhang et al. (2019)
	Elegant-crested tinamou, European quail and European Jackdaw	Stoessel & Fischer (2012)
	Quail	Andrada et al. (2013), Reilly (2000)
	Northern Lapwing	Nyakatura et al. (2012)
	Guinea fowl	Kambic, Roberts & Gatesy (2014)
	Ostriches	Zhang et al. (2017), Zhang et al. (2018)
	Pigeon	Cracraft (1971)
	Black-billed magpie	Verstappen, Aerts & van Damme (2000)
Hindlimb muscle function	Mallard	Taylor-Burt & Biewener (2020), Biewener & Corning (2001)
Webbed foot kinematics	Mallard	This study
Toe vertical displacement	Mallard	This study
Effect of speed on the toe joint angle	Mallard	This study

## Materials and Methods

### Animals

Four 24-month-old free-ranging male mallards, marked No. 1 to No. 4, were randomly selected from a professional mallard farm in Zhejiang Province, China, as the sample for this trial. Their mean body weight was 12.75 ± 43.30 g (expressed as mean ± SD of body weight). Four mallard ducks were examined with hip heights of 0.06, 0.07, 0.07, and 0.075 m, respectively. The four samples individually lived in custom-made duck cages during the trial and were given sufficient water and food to ensure a natural and healthy living condition for the mallard. Ethical approval was given by the Animal Experimental Ethical Inspection, Jilin University (reference No. SY202206100). After the test, all the mallard samples were healthy and returned to the farm.

The mallards were trained to walk on the treadmills for a fortnight, four times a week for 30 min each time, to get them adapted the rhythm of the treadmills during the trials. Some of the mallard’s wing feathers were removed without affecting the locomotion of the hind limbs to prevent the mallards from flying away during the trials and to enable the high-speed camera to capture clear marker points. Since the adhesive markers are too large for the mallard toe and tend to fall off, black markers were used to blacken the mallard joints as marker points. Thus, the mallard toes moved more naturally, whereas a clear and accurate video of the joints that is easy to process was obtained.

**Figure 1:**
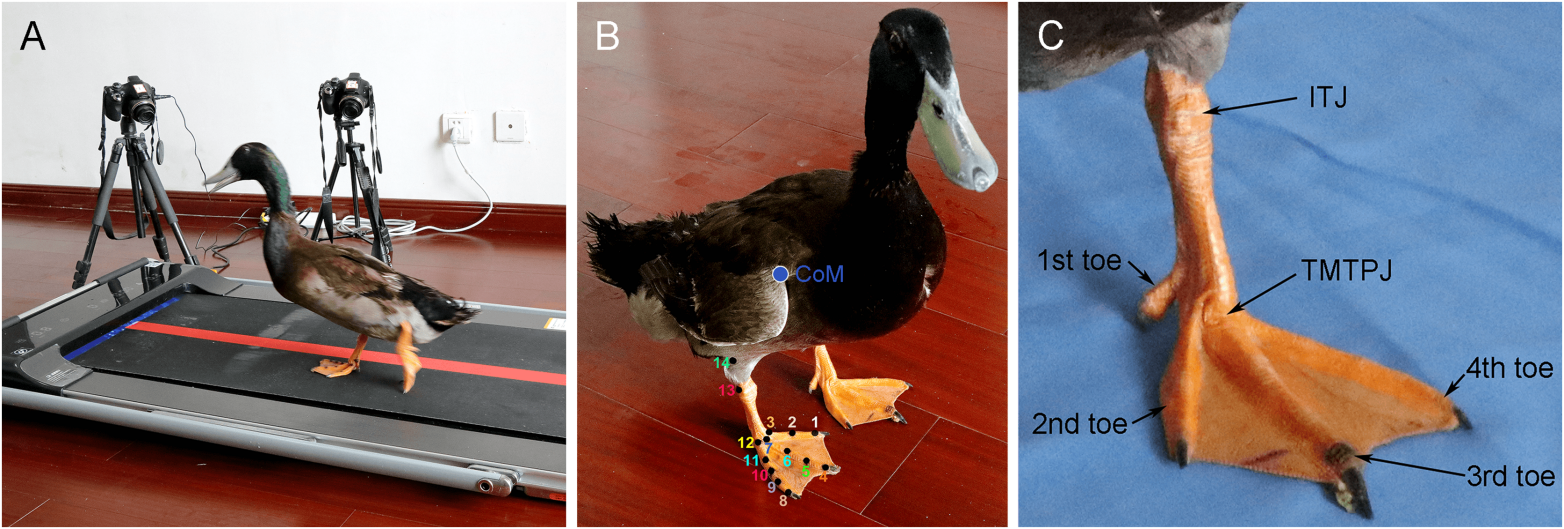
Test environment and marker positions. (A) Layout of the equipment. (B) Marker locations in the right foot of the mallard. CoM, centre of mass. (C) Joint and toe locations in the left foot of the mallard. 3D coordinates of these landmarks were used to determine the following 3D angles: the toetarsometatarso-phalangeal joint angle (TMTPJ angle, 6-7-13), the intertarsal joint angle (ITJ angle, 7-13-14), the α joint angle (2-3-7-6) between the second toe and the third toe, and the β joint angle (6-7-12-11) between the third toe and the fourth toe.

The construction of the test environment and the marker positions are illustrated in Fig. 1. The locomotion of the toes of the mallard was primarily completed by the second toe, the third toe and the fourth toe. Thus, the joint points on the three toes of the right foot of the mallard were marked: the second toe of the mallard has three marker points in total, namely, the dorsal ridge of the toenail (marker point 1), the first phalanx and the second interphalangeal joint (marker point 2), and the second TMTPJ (marker point 3). There were four marker points in the third toe, including the dorsal ridge of the toenail (marker point 4), the second phalanx and the third interphalangeal joint (marker point 5), the first phalanx and the second interphalangeal joint (marker point 6), as well as the third TMTPJ (marker point 7). There were five marker points in the fourth toe, which comprised the dorsal ridge of the toenail (marker point 8), the third segment of the phalanx and the fourth segment of the interphalangeal joint (marker point 9), the second segment of the phalanx and the third segment of the interphalangeal joint (marker point 10), the first segment of the phalanx and the second segment of the interphalangeal joint (marker point 11), as well as the fourth TMTPJ (marker point 12). Furthermore, there were ITJ (marker point 13) and tibiotatarsal bones (marker point 14).

### Kinematic test

The test site was selected in the laboratory, with good ventilation and sanitary conditions. A treadmill was selected for the test. The speed was accurate and adjustable through remote control, thus providing the speed step required for the test. The duck rope was used to guide the mallard to walk steadily and evenly on the treadmill with both feet, and the food temptation incentive was provided in front of the treadmill. If the mallard showed signs of reluctance to move, the experiment was immediately terminated. Kinematics software requires at least two cameras with an arrangement angle greater than 60 degrees to restore 3D space. Accordingly, two high-speed cameras (Casio Exilim EX-FH25; Casio, Tokyo, Japan) were employed for 120 Hz video recording and arranged respectively in the right front and right sides of the treadmill to ensure that the treadmill and mallards in motion can reveal the best vision of the two cameras. The respective frame was taken as a digital image with a resolution of 640 * 480 pixels (high-speed cameras), and four mallards were tested in sequence. Prior to the test, a 16-point calibration frame was shot for three-dimensional (3D) coordinate calibration, and the calibration video was recorded at the same angle as the motion video. Accordingly, all camera positions should be kept stable in the video recording process till the motion video and calibration video were completed. The calibration video would be shot again for any changes in the video recording process.

The speed range of this test was 1.0–5.0 km/h, and the speed step was set at 0.5 km/h. Thus, there were nine speeds in total, and no less than five valid videos were recorded at the respective speed. At the speed of 5.5 km/h, the mallards flew with their wings or move backward relative to the treadmill, whereas all the mallards reached a speed of 5.0 km/h.

### Data processing

After the data were processed using Simi-Motion kinematics software (Simi Reality Motion Systems GmbH, Unterschleißheim, Germany), the video import software with uniform motion speed and good condition was selected for tracking and analysis. At least three complete stride cycles were processed for each mallard at the respective speed. The spatiotemporal parameters of a total of 108 strides were analyzed (Table 2). The mechanical-energy fluctuation of the centre of mass of each mallard was calculated from the data derived from Simi-Motion by tracking a point corresponding to the hip joint (Nudds et al., 2010). After the data were exported, the mapping was completed using Excel (Microsoft, Redmond, WA, USA) and Origin Pro 8.5 (OriginLab Corporation, Northampton, MA, USA) and then processed using an FFT filter at a cut-off frequency of 5 Hz to ensure the originality and accuracy of key information (Zhang et al., 2019). %Congruity is calculated using the product of instantaneous changes (high-video frames) between E_p_ + E_kv_ and E_kh_ of the centre of mass. All cases where the product is greater than zero, *i.e*., when the two energies are consistent, are added up and reported as a percentage of the overall frame (Nyakatura et al., 2012). Ideally, %Congruity would be 0% for a walking trial and 100% for a running trial (Ahn & Biewener, 2004). Andrada et al. (2013) defined walking for %Congruity values <50 and running for %Congruity values >50. Moreover, the results and regularity of the spatiotemporal parameters (*e.g*., stride length, stride duration, stance phase duration, swing phase duration, and duty factor), locomotion gait, continuous joint angle, instantaneous joint angle, pattern of toes and webbed motion, and toe vertical displacement of the mallard’s toe were analyzed. Both linear (y = ax + b) and non-linear (y = ax^b^) regressions were performed using Origin Pro software for spatiotemporal parameters to illustrate trends in the data on the graphs. To select the best-fit curve, the mean-corrected R^2^ from the non-linear regression was compared to the adjusted R^2^ from the linear regression (Hancock, Stevens & Biknevicius, 2007). The effect of the speed on TMTPJ and ITJ of the mallard during the stance phase duration was examined using One-Way ANOVA. F test was performed to compare the means at the significance level of 0.05, and Bonferroni adjustment was also employed for suitable corrections.

**Table 2:** The average and standard deviation of significant parameters in terms of the walking and grounded running gait of mallards.

Gait parameters	Walking	Grounded running
Number of trials	160	
Number of statistics analysis	53	55
Average speed (m/s)	0.60 ± 0.19	1.26 ± 0.23
Stride duration (s)	0.59 ± 0.12	0.37 ± 0.05
Stride length (m)	0.34 ± 0.06	0.46 ± 0.04
Stance phase duration (s)	0.45 ± 0.12	0.23 ± 0.04
Swing phase duration (s)	0.15 ± 0.02	0.14 ± 0.02
Duty factor	0.74 ± 0.06	0.62 ± 0.04

**Note:**

Values are means ± SD.

## Results

### Changes of spatiotemporal parameters

Table 2 lists the average and standard deviation of significant parameters in terms of the walking gait of mallards.

**Figure 2:**
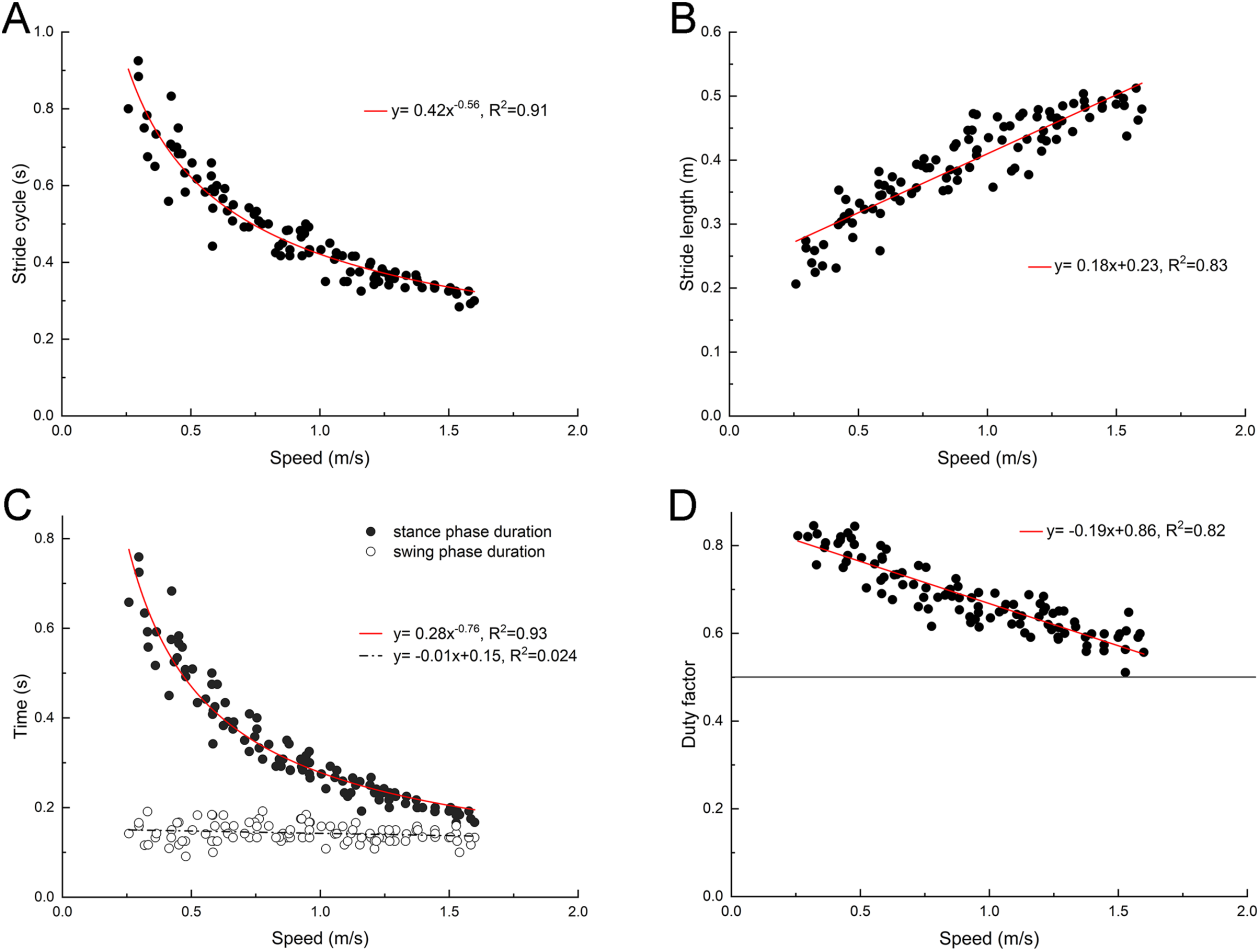
Changes of basic spatiotemporal gait parameters with speed. (A) Stride cycle. (B) Stride length. (C) Stance and swing phase duration. (D) Duty factor. Model for best fit curvilinear regression and coefficient of determination (R^2^) provided in the respective graph.

The back ridge of the third toenail of the right foot of the mallard (marker point 4) was used to determinate the instant of lift-off and touch-down, and the change trend of stride duration, stride length, swing phase duration and stance phase duration was determined. As depicted in Fig. 2, with the increase of the treadmill speed, the stride length of the mallard tended to increase, whereas the stride duration tended to decrease. In the stride cycle, the stance phase duration tended to decrease, and the swing phase duration did not vary significantly. Thus, the duty factor tended to decrease as well. However, no data had the duty factor of less than 0.5.

### Instantaneous changes of the joint angles

The regularity between the TMTPJ and ITJ and the velocity was analyzed at the right toe of the mallard in the touch-down (0%), mid-stance (50%), and lift-off (100%) moments.

**Figure 3:**
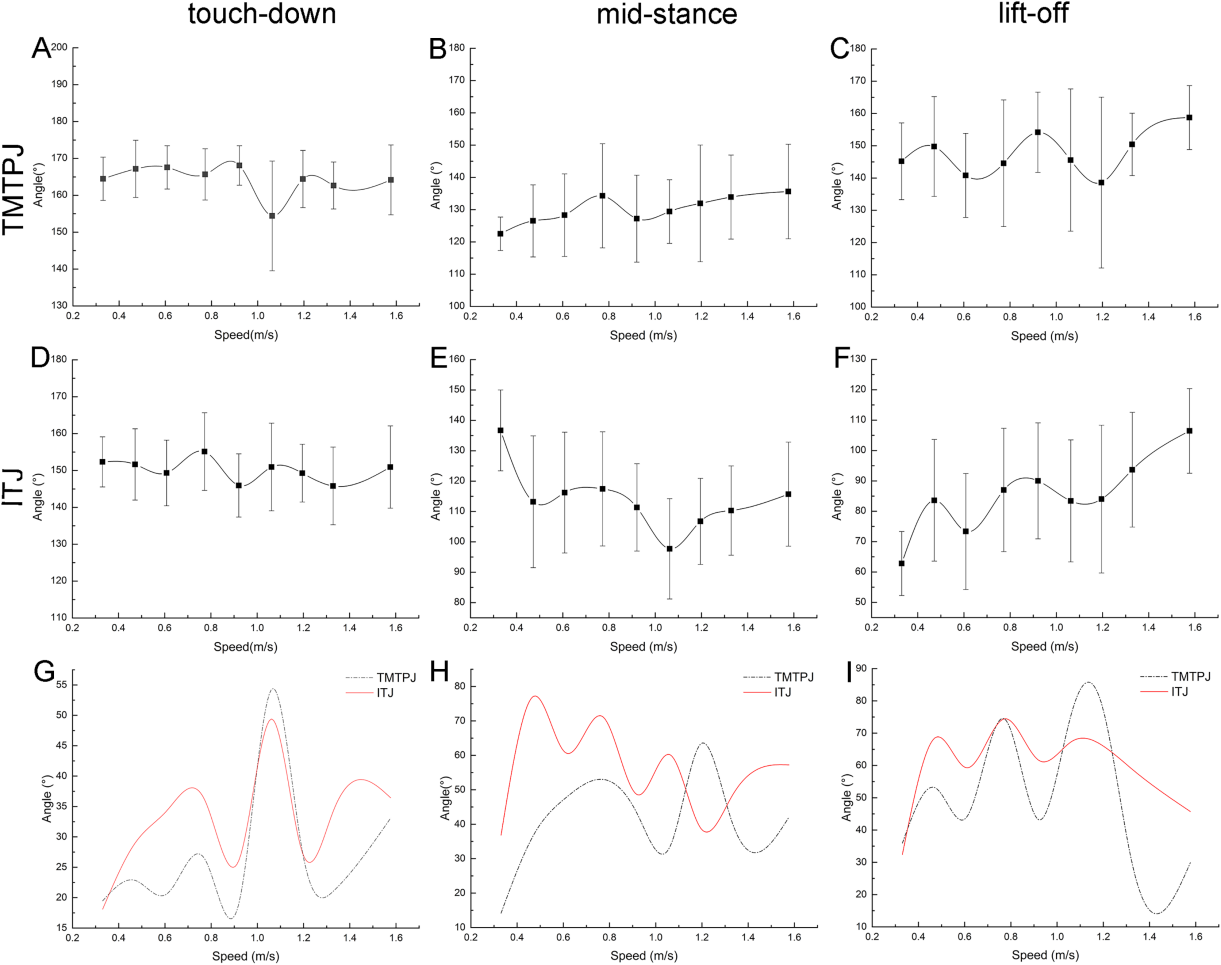
Changes of instantaneous joint angles during the duration of the stance phase with increasing speed (108 stride cycles totally). (A–C) Changes of the TMTPJ angle at touch-down, mid-stance, and lift-off, respectively. (D–F) Changes of the ITJ angle at touch-down, mid-stance, and lift-off, respectively. (G–I) Changing ranges compared between the TMTPJ angle and the ITJ angle at touch-down, mid-stance, and lift-off, respectively.

As depicted in Figs. 3A–3C, with the increase of the speed, the TMTPJ of the mallard had a slight effect at the touch-down, mid-stance, and lift-off moments. As depicted in Figs. 3D–3F, with the increase of the speed, the ITJ of the mallard was significantly affected in the mid-stance and lift-off moments, where the angle at the mid-stance decreased first and then increased, and the angle at lift-off tended to increase. As depicted in Figs. 3A and 3D, the trend of TMTPJ angle and ITJ angle was nearly similar at the touch-down moment, whereas the change of ITJ angle was earlier than that of TMTPJ angle. The change range of the angle of the TMTPJ and ITJ at the respective speed at the touch-down, mid-stance and lift-off moments was obtained (Figs. 3G–3I), and the image of the ITJ angle was mostly above the TMTPJ angle.

Table 3 lists the effects of speed on changes in the TMTPJ and ITJ angles that were tested through significance analysis. It is evident that while the change in speed has little effect on the TMTPJ angle changes, it has a significant effect on the ITJ angle, particularly during mid-stance and lift-off. The reason for this phenomenon may be that mid-stance is considered as the moment of articulation, and mallards vary the joint angle in a wider range to accommodate the leg-foot stance.

**Table 3:** Significance analysis of the influence of speed on joint angles during the duration of the stance phase.

Speed (km/h)	TMTPJ (°)									ITJ (°)								
	Touch-down			Mid-stance			Lift-off			Touch-down			Mid-stance			Lift-off		
	Max	Min	Means ± SD	Max	Min	Means ± SD	Max	Min	Means ± SD	Max	Min	Means ± SD	Max	Min	Means ± SD	Max	Min	Means ± SD
1	173.5	154	164.5 ± 5.9^a^	128.8	114.7	122.5 ± 5.2^b^	164.6	128.7	145.2 ± 11.9^abc^	160.5	142.4	152.3 ± 6.8^ab^	155.9	119.1	136.7 ± 13.3^a^	82.7	50.3	62.8 ± 10.5^d^
1.5	176.8	153.9	167.2 ± 7.8^a^	150.1	113.2	126.5 ± 11.2^ab^	172.9	119.8	149.8 ± 15.5^abc^	165.5	136.5	151.6 ± 9.7^ab^	155	77.8	113.2 ± 21.7^b^	129.9	61.3	83.6 ± 20^bc^
2	175.3	154.7	167.6 ± 5.9^a^	150.6	103	128.3 ± 12.8^ab^	160	116.1	140.8 ± 13^bc^	164.5	130.2	149.3 ± 8.9^ab^	146.5	85.7	116.2 ± 19.9^b^	110.3	51	73.4 ± 19.1^cd^
2.5	174	147.7	165.7 ± 6.9^a^	164.6	111.5	134.3 ± 16.1^a^	172.9	98.4	144.6 ± 19.6^bc^	167.1	131.1	155.1 ± 10.5^a^	148.6	77.4	117.4 ± 18.8^b^	124.5	50	87 ± 20.3^bc^
3	177.4	158.2	168.1 ± 5.4^a^	157.5	114.6	127.2 ± 13.5^ab^	174.2	131.1	154.2 ± 12.4^ab^	159.2	133	145.9 ± 8.6^b^	138.7	90.2	111.3 ± 14.4^bc^	121.6	59.9	90 ± 19.1^b^
3.5	174.3	120	154.4 ± 14.9^b^	142.4	108.8	129.4 ± 9.9^ab^	168	91.8	145.6 ± 22^abc^	174.8	125.5	150.9 ± 11.9^ab^	128.6	68.4	97.7 ± 16.5^c^	111.8	44.4	83.4 ± 20.1^bc^
4	176.8	149.3	164.4 ± 7.8^a^	166.7	103.3	131.9 ± 18.1^ab^	168.5	90.1	138.6 ± 26.5^c^	162.5	135.2	149.3 ± 7.8^ab^	126.7	87.9	106.7 ± 14.2^bc^	120.8	54.6	84 ± 24.3^bc^
4.5	174.9	153.8	162.7 ± 6.4^a^	155.7	114.8	133.9 ± 13^ab^	164.7	136.2	150.4 ± 9.7^abc^	165.1	130.8	145.8 ± 10.5^b^	133.1	85.1	110.3 ± 14.7^bc^	123.3	64.7	93.7 ± 18.9^ab^
5	175.8	142.7	164.2 ± 9.5^a^	154.9	112.8	135.6 ± 14.7^a^	172.7	142.8	158.7 ± 9.9^a^	173.2	136.7	150.9 ± 11.2^ab^	142.1	84.9	115.7 ± 17.1^b^	122.1	76.4	106.5 ± 13.9^a^

**Note:**

Values were means ± SD. Statistically significant effects of different speeds are indicated by superscripted letters (*P* < 0.05).

### Continuous changes of the joint angles

**Figure 4:**
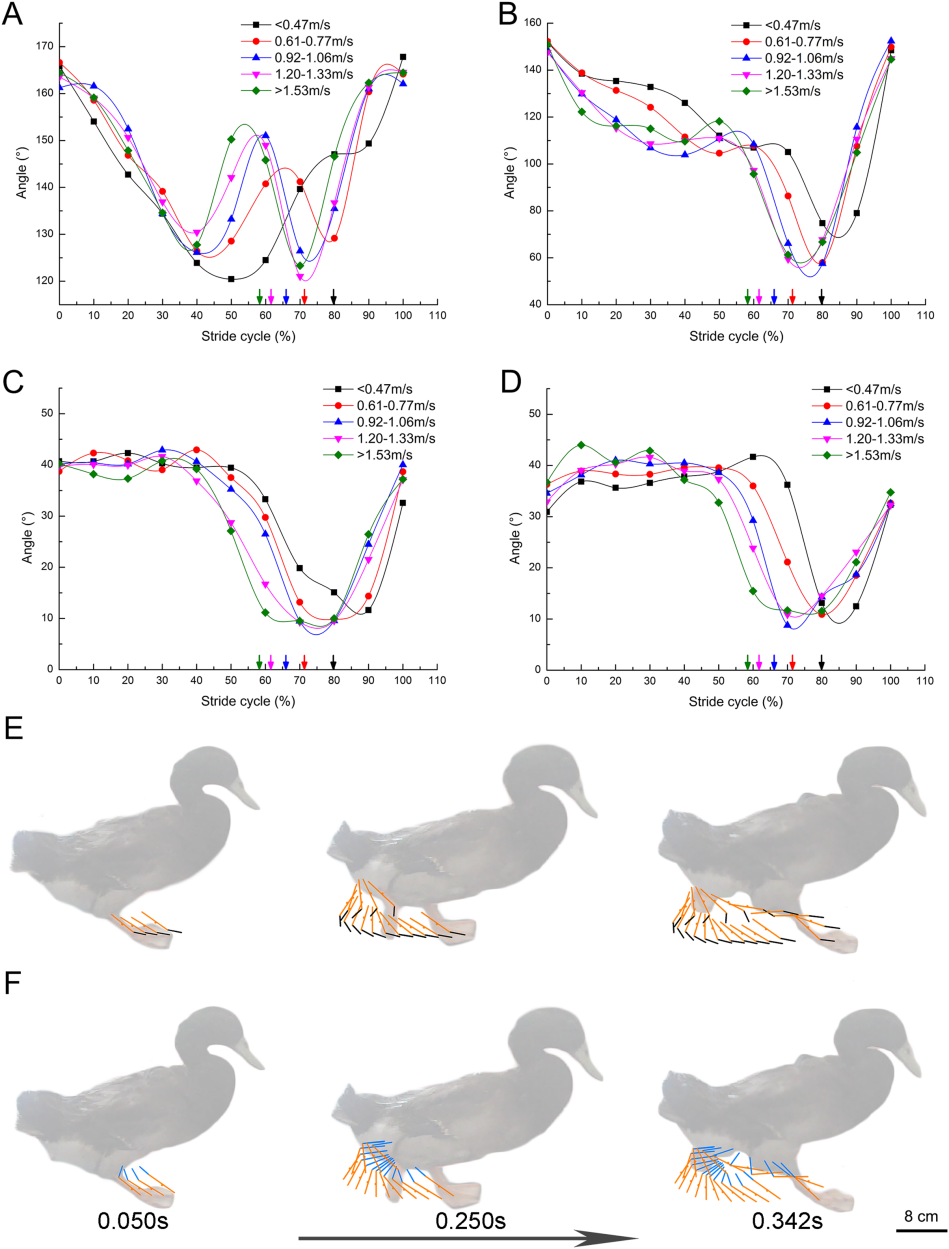
Continuous changes of joint angles with speed (108 stride cycles totally). (A–D) Changes of the TMTPJ angle, the ITJ angle, the α joint angle and the β joint angle in a stride cycle, respectively. The arrows represent the split points between stance and swing phase duration. Changes in (E) the TMTPJ angle and (F) the ITJ angle are illustrated by the stick figures, and the time difference between the lines was 1/60 s.

The change of TMTPJ angle in a complete stride cycle is shown in Fig. 4A. The change of TMTPJ angle is in the form of letter W between 120° and 170°. In addition, the curve moves to the left with the increase of the speed, suggesting that the increase of the speed will make the TMTPJ angle change ahead of time in a stride cycle. The changes of TMTPJ angle are presented in stick Fig. 4E. The change of the ITJ angle during the stride cycle is shown in Fig. 4B. The change of the ITJ angle is in the range of 50° to 150°. It first decreases gradually, then increases slightly, then decreases rapidly, and finally increases rapidly to the initial angle. The change range of the angle during the stance phase duration is smaller than that during the swing phase duration. In addition, the curve moves to the left with the increase of the speed, thus suggesting that the increase of the speed will also make the ITJ angle change ahead of time in a stride cycle, and the stance phase duration will gradually shorten to a certain extent, such that the duty factor will decrease with the increase of the speed. The changes of ITJ angle are presented in stick Fig. 4F.

The α joint angle between second toe and the third toe and the β joint angle between third toe and fourth toe were defined, and the changes of the α and β joint angle with the increased speed in a stride cycle were studied (Figs. 4C and 4D). α and β joint angle was first kept to be essentially constant (α joint angle roughly 40°, β joint angle roughly 37°), then decreased rapidly, and subsequently increased. With the increase of the speed, both the α and β joint angle curves shifted to the left, thus revealing that an increase in speed leads to the change of α and β joint angles earlier in a stride cycle and result in the earlier close of three toes. The angle of the three toes of the mallard remained at the maximum in the stance phase duration of the stride cycle, tended to close to the minimum angle in the first half of the swing phase duration, and progressively splayed in the second half of the swing phase duration till the maximum angle at touch-down again.

### Changes of the toe vertical displacement

**Figure 5:**
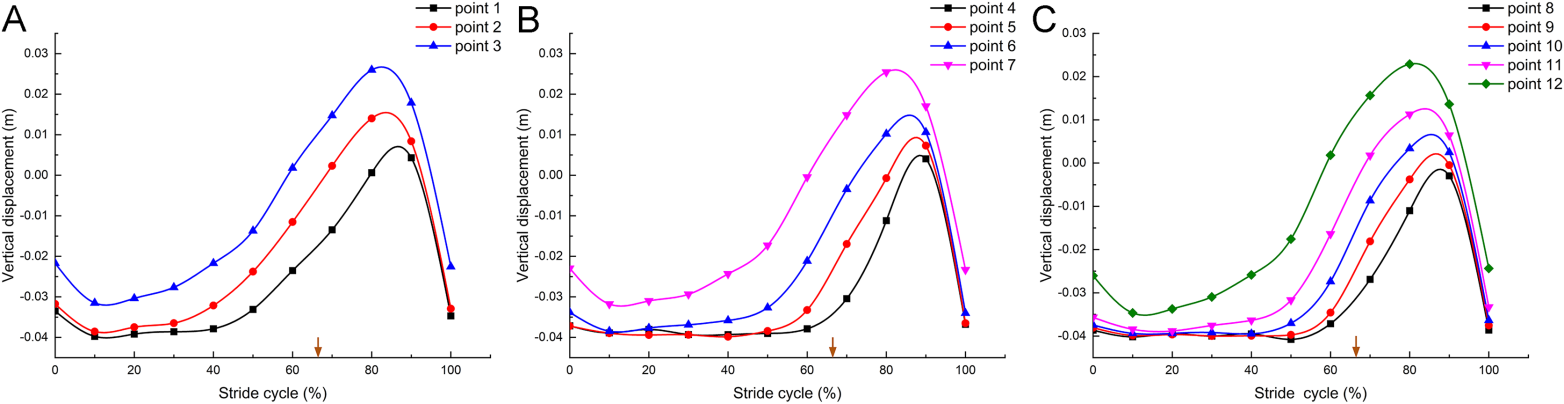
Vertical displacement changes of the markers in the mallard’s toes in a stride cycle (speed = 3.0 km/h, 12 stride cycles totally). Vertical displacement changes of the second (A), third (B) and fourth (C) toes. The arrows represent the split points between the stance and the swing phase duration.

In a complete stride cycle (Fig. 5A), the vertical heights of marker point 1, marker point 2 and marker point 3 on the second toe of the mallard first decreased briefly and then stabilized at a certain height. Subsequently, the vertical height of marker point 3 began to rise, and those of marker point 2 and marker point 1 followed closely. Next, the vertical heights tended to decrease and nearly overlapped after reaching the highest point, whereas the height of marker point 3 was higher than that of marker point 2, and the height of marker point 1 was the lowest in the whole stride cycle. Figures 5B and 5C are nearly consistent with the above. Furthermore, the vertical displacement changes of the joint points on the second toe, the third toe, and the fourth toe presented very similar motion patterns.

## Discussion

### Locomotion gait of the mallard

**Figure 6:**
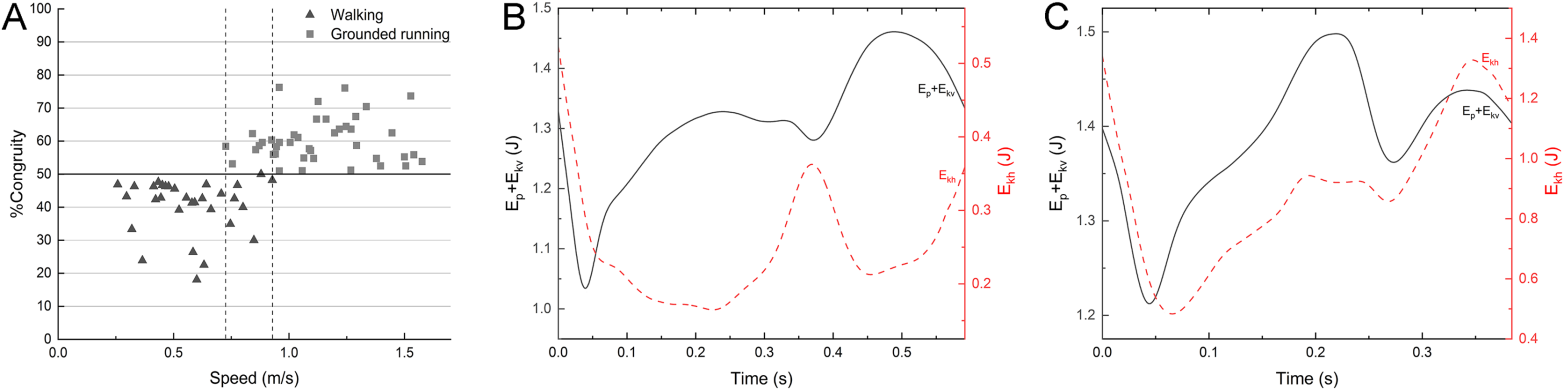
%Congruity *vs* speed (A) and representative curves of E_p_ + E_kv_ and E_kh_ for a walking gait (B) and a grounded running gait (C). %Congruity values lower up to 50 are often related to vaulting mechanics, while those larger than 50 are interpreted as bouncing mechanics. The gait of (B) had a speed of 0.63 m/s and %Congruity of 22.5. The gait of (C) had a speed of 1.24 m/s and %Congruity of 76.1. E_kh_, horizontal kinetic energy; E_p,_ gravitational potential energy; E_kv,_ vertical kinetic energy.

The mallard did not have a duty factor less than 0.5 in the test data on the laboratory treadmill. Even though the duty factor is close to 0.5 at the highest speed, although we have used various ways to stimulate the mallard, the mallard will fly with their wings or cannot keep up with the treadmill at the speed of 5.5 km/h. The percentage of congruity analysis is shown in Fig. 6A. Between 0.73 and 0.93 m/s, the gait experienced a transition from walking to grounded running, with no significant changes in spatiotemporal parameters. At speeds between 0.26 and 0.73 m/s, mallards adopt a walking gait is mainly used and conform to vaulting mechanics (Fig. 6B). At speeds between 0.93 and 1.6 m/s, mallards adopt a grounded running gait conform to bouncing mechanics (Fig. 6C), where the body bounces while maintaining continuous contact with the ground. Consistently, the mallard and Indian runner duck of the three varieties of ducks tested by Usherwood, Szymanek & Daley (2008) have a running gait at the highest speed (albeit predominantly “grounded running” without aerial phases). Interestingly, not all ducks have a running gait (*i.e*., aylesbury duck, Usherwood, Szymanek & Daley, 2008), which means that there are slight differences in the land gait even among similar species. The duty factor of the barnacle geese (Nudds et al., 2010) was negatively correlated with the speed, whereas it did not drop below 0.5, meanwhile, the gait of a barnacle goose appears to conform to the classical pendulum mechanics based model of walking, with E_kh_ out-of-phase E_p_ + E_kv_ at the highest speed, thus suggesting that the barnacle geese did not change into a running gait. Animals often switching back and forth between different environments are challenged by using the same propulsion structure and muscles in media with various physical properties (Taylor-Burt & Biewener, 2020). In the land locomotion, similar to the barnacle goose, the mallard has a staggered step with its feet facing inward due to the lateral and backward distribution of the tibia and tarsus in the position during standing (Provini et al., 2012). In other words, the body in the walking process should rotate and swing around the supporting leg to transfer the center of gravity to the supporting leg (Daanje, 1951). However, such gait will lead to lateral momentum, increasing the instability during locomotion. With the further increase of the speed, this lateral momentum will increase the swing amplitude of the mallard’s body, thus increasing unnecessary energy consumption, which will hinder its longitudinal displacement. The research on ringed teal (*Callonetta leucophrys*) of Provini et al. (2012) suggested that the shape of the musculoskeletal system and its function will be affected under the need for the hind limbs to play different roles. The semi-aquatic life style of paddling birds corresponds to morphological adaptation. Thus, the land tottering gait of the mallard may be a compromise for the evolution of swimming (Provini et al., 2012).

The mallard adapts to the increase of the speed by shortening the stance phase duration and increasing the stride length, while the swing phase duration is basically unchanged. Accordingly, the stride duration and duty factor show a decreasing trend. The swing phase duration is basically unchanged that differs from the findings of Abourachid (2001). When Abourachid (2001) studied rhea, kiwi, paleognatiforms, Indian runner ducks, mallard, quail and guinea fowl, and found the stance phase duration would decrease with the increase of the relative speed, while the swing phase duration was almost constant, except that the swing phase duration of the mallard would increase slightly with the increase of the relative speed. Abourachid (2001) thinks that the long swing duration in the mallard may be due to an increase in waddling movements when it moves more quickly.

### Ground contact posture of the mallard toes

**Figure 7:**
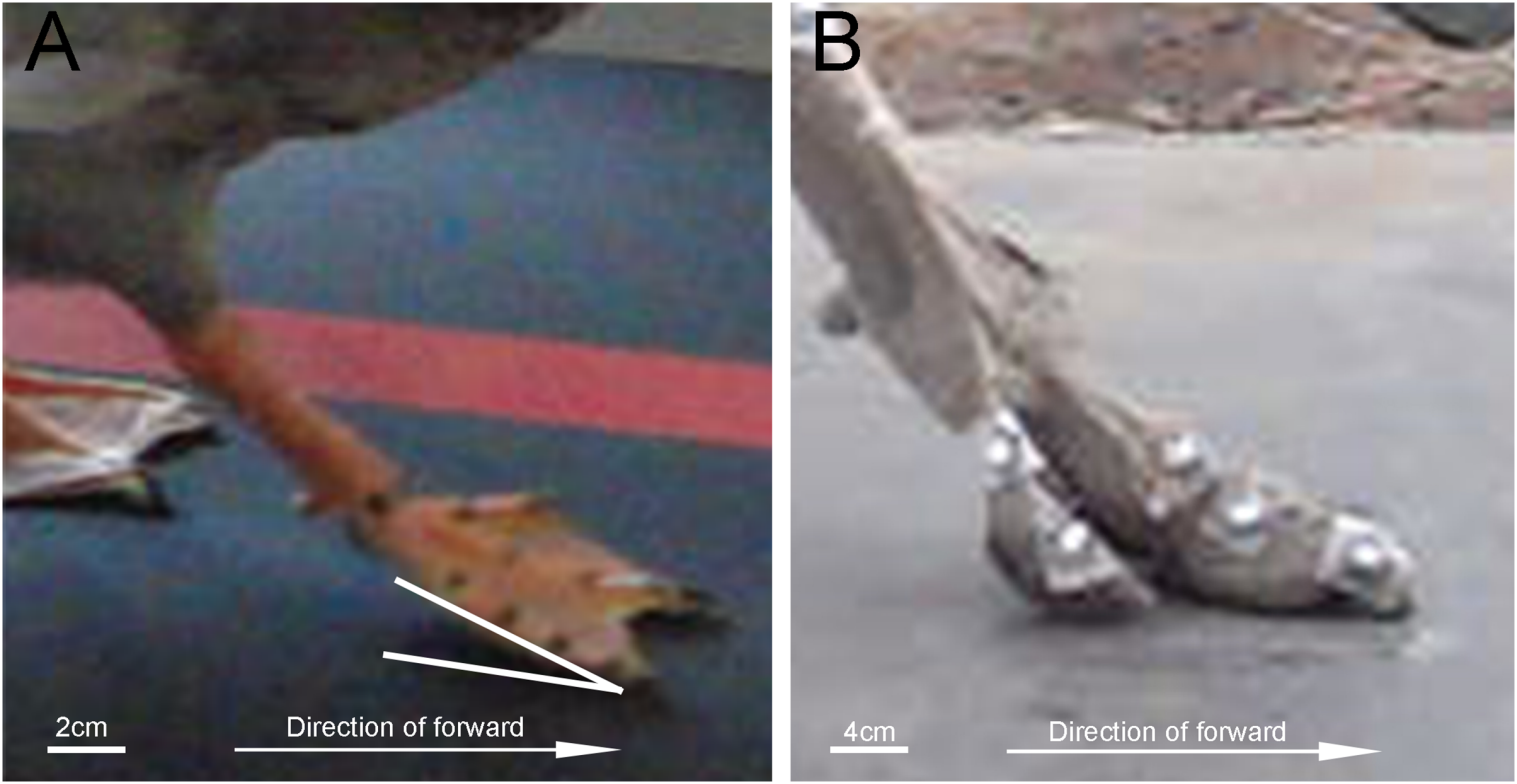
Comparison of the lateral view of touch-down between mallard foot and ostrich foot. (A) The right foot of the mallard foot (The white line represents the angle between the mallard’s foot and the ground). (B) The right foot of the ostrich foot.

In a complete stride cycle, the distal phalanx of the second, third and fourth toes of the mallard first touches the ground, and the proximal phalanx touches the ground instantaneously (Fig. 5, the first 10% of the stride cycle). In ostrich feet, the first and second toes degenerated, and only two toes were retained (Alexander, 1985). When the whole foot touches the ground, it touches the ground instantly (Fig. 7). Its running speed and weight are far more than those of birds such as mallards. The permanent elevation of TMTPJ will provide cushioning and store energy during locomotion. Ducks are unlikely to be used exclusively for elastic energy storage and recovery. Their legs are relatively short and flat, and their tendons are thick, which may reflect some compromises related to the evolutionary pressure of swimming (Usherwood, Szymanek & Daley, 2008). In some four-toed birds (*e.g*., pigeons (Cracraft, 1971) and magpies (Verstappen, Aerts & van Damme, 2000)), the first toe touches the ground before the front toe in their gait, and then the heel surface contacts the ground again. Thus, the phenomenon that the tip of a mallard’s toe touches the ground first and then the heel touches the ground instantaneously may be caused by the synergetic effect with the first toe.

When the foot leaves the ground, it starts from the proximal phalanx to the distal phalanx, consistent with Zhang et al. (2018) finding that the vertical displacement of ostrich’s TMTPJ on sand ground and hard ground decreases first and then increases. It is therefore revealed that the foot rotates around the TMTPJ when it leaves the ground, so the TMTPJ angle increases, which explains the reason why the TMTPJ angle curve in Fig. 4A increases for the first time. In the whole process of lifting off the ground, the vertical height of the proximal joint point is greater than the vertical height of the distal joint point in the curve ascending phase, thus suggesting that the foot is in a drooping state during the swing phase duration of the elevation stage; The vertical heights of the proximal and distal joint points begin to overlap at the curve descent stage, which indicates that the mallard starts to adjust its feet and gradually approaches the level to undertake the next touch-down in the swing phase duration of the descent stage.

### Webbed foot motion system of the mallard

As revealed by the result of the significance analysis of the instantaneous joint angle, with the increase of the speed, the TMTPJ of the mallard is slightly affected, whereas the ITJ is more significantly affected at the moment of mid-stance and lift-off. The effect of the moment of lift-off is greater than mid-stance. Zhang et al. (2019) noted that the increase in speed significantly affected the ITJ of pheasants at the mid-stance. They have suggested that the possible reason for the above result was that the mid-stance served as the connection time, and pheasant changed the ITJ angle in a wider range to adjust the leg posture. It is speculated that due to the difference in walking posture between the pheasant and the mallard, the adjustment of ITJ of the mallard is more delayed due to its waddling locomotion.

The analysis of the continuous joint angle indicated that the TMTPJ angle of the mallard was obtuse in a complete stride cycle when it touched the ground, and then the metatarsal bone started to rotate around the TMTPJ, thus resulting in the reduced TMTPJ angle. The ITJ angle was reduced after the foot started to leave the ground; with the metatarsal bone elevation, the TMTPJ angle tended to increase to the superior angle and decrease to the obtuse angle before the next touch-down. The ITJ angle of the mallard (100°) had a greater change amplitude than the TMTPJ angle (50°), thus suggesting that the mallard primarily change their hindlimb postures or gaits by retracting and protracting ITJ rather than TMTPJ with increasing speed. With the increase of the speed, the ITJ angle and the TMTPJ angle changed in advance, whereas the overall change trend remained unchanged, thus confirming the shortening of the stance phase duration.

The reduction of α and β joint angles between the toes of the mallard in the first half of the swing phase duration is probably explained as follows: the mallard’s web reduces the air/water resistance when moving on the ground/aquatic, and the mechanism may be the joint pulling effect of the flexor tendons and ligaments of the mallard feet. In the second half of the swing phase duration, the ankle joint and the toes start to extend. Since ankle extension unstrains the toe flexor tendons, toe extension can occur passively (Verstappen, Aerts & van Damme, 2000). With the α and β joint angles gradually increased, the web to splay to receive the next touch-down with the maximum ground contact area, thus suggesting the sink resistance of the mallard to a certain extent. α and β joint angles changed with similar trends at the same speed and with similar trends with the increase of the speed, thus suggesting that the second and fourth toes in relative to the change of the third toe were highly synchronized. Furthermore, the decrease/increase of α and β joint angles simultaneously the web between the toes to close/splay, and the web also limited the maximum angle of the two joint angles, thus revealing that the webbed foot plays a certain role in the ground motion process as a system.

Nonetheless, the above-described results should be carefully explained, and some limitations should be considered. The high similarity between the second and fourth toe movements in mallard ducks cannot be explained in accordance with the present kinematic results. And also value of mechanical-energy fluctuations in the centre of mass calculated using an external mark are likely to be inaccurate. Subsequent anatomical analysis research may provide answers to these question.

## Conclusions

Mallards adopt a grounded running gait at speeds of 0.93–1.60 m/s and conform to bouncing mechanics. At lower speeds a walking gait is mainly used and is consistent with the vaulting mechanics. The continuous curve of change in joint angle shifts to the left with the increase of the speed, thus confirming that the stance phase duration is shortened. The mallard responds to the increase in speed mainly by adjusting the ITJ, instead of the TMTPJ. The distal phalanx of the toe contacts the ground first during touch-down, and then the proximal phalanx touches the ground instantaneously. When it leaves the ground, the proximal phalanx starts to leave the ground sequentially. Moreover, with the decrease of the interphalangeal α and β joint angles, the foot web tends to close and rapidly recover before the next touch-down. The high degree of synchronization of the mallard’s toes II, III and IV is considered an integrated system, and the webbed foot serves as a wholed system that plays a role in the adjustment of speed in the ground locomotion. This article provides experience and technical reference for the research on the toe kinematics of bipedal walking birds.

## Supplemental Information

10.7717/peerj.15362/supp-1 Supplemental Information 1 Raw data.

10.7717/peerj.15362/supp-2 Supplemental Information 2 Significance analysis source file.

10.7717/peerj.15362/supp-3 Supplemental Information 3 Raw data specification.

10.7717/peerj.15362/supp-4 Supplemental Information 4 Mallard walking on the treadmill.

10.7717/peerj.15362/supp-5 Supplemental Information 5 Author Checklist.

## Additional Information and Declarations

### Data Availability

The following information was supplied regarding data availability: Raw data is available in the Supplemental Files.
