# Supplementary material for: Effects of the speed on the webbed foot kinematics of mallard (Anas platyrhynchos)
Source: PeerJ. 2023 May 15;11:e15362. doi: 10.7717/peerj.15362 (PMC10194065; doi:10.7717/peerj.15362)
Supplement: Supplemental Information 3 [file peerj-11-15362-s003.docx]

### Notes: How to use the raw data

#### Figure 2

We analyzed the spatio-temporal parameters of a total of 108 strides. After the data were exported, the mapping was completed using Excel (Microsoft, Redmond, WA, USA) and Origin Pro 8.5 (OriginLab Corporation, Northampton, MA, USA), and processed using an FFT filter at a cut-off frequency of 5 Hz to ensure the originality and accuracy of key information (Zhang et al., 2019).

The actual speed of the mallard was as the abscissa, and stride cycle, stride length, stance and swing phase duration and duty factor were taken as the ordinate to draw scatter plots corresponding to Fig 2. A, B, C and D, respectively.

Both linear (y = ax + b) and non-linear (y = ax^b^) regressions (Hancock et al., 2007) were performed using Origin Pro software for spatiotemporal parameters to illustrate trends in the data on the graphs. For each variable pair, the adjusted R^2^ from the linear regression was compared with mean-corrected R^2^ from the non-linear regression to determine the best fit curve.

#### **Figure 3**

We took the average speed of mallard as the abscissa, and the average angle of TMTPJ and ITJ at the touch-down, the mid-stance, the lift-off as the ordinate to draw the Bessel curve with error bar, corresponding to Fig 3. A, B, C, D, E and F, respectively.

We took the average speed of mallard as the abscissa, and the extreme value (max-min) of TMTPJ and ITJ at the touch-down, the mid-stance, the lift-off as the ordinate to draw the Bessel curve, corresponding to Fig 3. G, H and I, respectively.

The effect of the speed on TMTPJ and ITJ of the mallard during the stance phase duration was examined using One-Way ANOVA. F test was performed to compare the means at the significance level of 0.05, and Bonferroni adjustment was also employed for suitable corrections.

#### **Figure 4**

In order to more intuitively and clearly show the changes of continuous joint angles under the 9 kinds of velocities, we combined them into 5 velocity intervals (<0.47 m/s, 0.61 - 0.77 m/s, 0.92 - 1.06 m/s, 1.20 - 1.33 m/s, >1.53m/s), respectively.

We took the stride cycle as the abscissa, and the average angle of TMTPJ, ITJ, α and β as the ordinate to draw the Bessel curve, corresponding to Fig 4. A, B, C and D, respectively. The arrows indicate the split points between stance and swing phase duration.

#### **Figure 5**

We took the stride cycle as the abscissa, and the vertical displacement of marker points on the second, third and third toes as the ordinate to draw the Bessel curve, corresponding to Fig 5. A, B and C, respectively. Among them, the second toe marker points were 1, 2, 3, the third toe marker points were 4, 5 ,6, 7, and the fourth toe marker points were 8, 9, 10, 11, 12. The arrows indicate the split points between stance and swing phase duration.

#### **Figure 6**

We drew a scatter plot as shown in Fig. 6 (A) with the green duck velocity as the abscissa and the %Congruity as the ordinate. %Congruity values lower up to 50 are often related to vaulting mechanics, while those larger than 50 are interpreted as bouncing mechanics.

We draw the Bessel plot as shown in Fig. 6 (B) and (C) with the stride time as the abscissa and E_p_+E_kv_ and E_kh_ as the ordinate. The gait of (B) had a speed of 0.63 m/s and %Congruity of 22.5. The gait of (C) had a speed of 1.24 m/s and %Congruity of 76.1.

CoM Z, vertical displacement of the centre of mass; CoM V_X_, x-directional velocity of the center of mass; CoM V_Y_, y-directional velocity of the center of mass; CoM V_Z_, vertical velocity of the center of mass. E_kh_, horizontal kinetic energy; E_p,_ gravitational potential energy; E_kv,_ vertical kinetic energy.
